# Superprotonic Conductivity in a Metalloporphyrin-Based SMOF (Supramolecular Metal–Organic Framework)

**DOI:** 10.3390/nano14050398

**Published:** 2024-02-21

**Authors:** Arkaitz Fidalgo-Marijuan, Idoia Ruiz de Larramendi, Gotzone Barandika

**Affiliations:** 1 Department of Organic and Inorganic Chemistry, University of the Basque Country (UPV/EHU), Barrio Sarriena s/n, 48940 Leioa, Spain; arkaitz.fidalgo@ehu.eus; 2 BCMaterials, Basque Center for Materials, Applications and Nanostructures, Barrio Sarriena s/n, 48940 Leioa, Spain

**Keywords:** proton conductivity, metalloporphyrins, SMOF, solid-state electrochemical devices

## Abstract

Metal–organic frameworks and supramolecular metal–organic frameworks (SMOFs) exhibit great potential for a broad range of applications taking advantage of the high surface area and pore sizes and tunable chemistry. In particular, metalloporphyrin-based MOFs and SMOFs are becoming of great importance in many fields due to the bioessential functions of these macrocycles that are being mimicked. On the other hand, during the last years, proton-conducting materials have aroused much interest, and those presenting high conductivity values are potential candidates to play a key role in some solid-state electrochemical devices such as batteries and fuel cells. In this way, using metalloporphyrins as building units we have obtained a new crystalline material with formula [H(bipy)]_2_[(MnTPPS)(H_2_O)_2_]·2bipy·14H_2_O, where bipy is 4,4′-bipyidine and TPPS^4−^ is the *meso*-tetra(4-sulfonatephenyl) porphyrin. The crystal structure shows a zig-zag water chain along the [100] direction located between the sulfonate groups of the porphyrin. Taking into account those structural features, the compound was tested for proton conduction by complex electrochemical impedance spectroscopy (EIS). The as-obtained conductivity is 1 × 10^−2^ S·cm^−1^ at 40 °C and 98% relative humidity, which is a remarkably high value.

## 1. Introduction

Proton conduction occurs ubiquitously in biological systems. Protons are the smallest ions in nature, so they are involved in many important processes, such as acid/base reactions, enzyme catalysis, or photosynthesis [1,2,3,4,5,6,7]. Proton circuits have been widely studied in biological systems as they are transported across membranes in bioenergetic systems [8,9,10]. In fact, due to its technological relevance in energy applications (such as water electrolyzers, fuel cells, and batteries), research on proton conductivity shifted decades ago toward solid-state conductors including polymers, ceramic oxides, composites, metal–organic frameworks (MOFs) and supramolecular metal–organic frameworks (SMOFs) [11,12,13,14,15].

MOFs and SMOFs, in particular, are very promising proton conductive materials [4,12,16,17,18,19,20]. MOFs and SMOFs are crystalline porous materials composed of metal nodes coordinated by organic ligands, and therefore a subclass family of coordination polymers. The first demonstration of proton conductivity in coordination polymers was reported by Kanda et al. in 1979 [21] for a porous material. More than two decades later, Nagao et al. observed proton conductivity in two Cu-based coordination polymers [22,23]. These studies were the re-starting point for further research on the application of coordination polymers as solid-state proton conductors. Since then, numerous studies have been focused on MOFs and SMOFs as these materials exhibit several advantages, such as tunable pore size in countless structures (by a combination of different metal ions and organic ligands), in-pore control of hydrophilicity or acidity, post-synthetic modification, and thermal and water stability [12,24,25,26,27,28,29].

Over the last years, numerous coordination polymer-based proton conductors have been reported, and the highest values of proton conductivity in MOFs are on the order of 10^−2^ to 10^−1^ S·cm^−1^ under high humidity conditions [30,31,32,33]. These values are comparable to those observed for the commercial perfluorosulfonate membrane (Nafion) [34,35]. Proton conduction has also been explored in protein-based materials, [36,37] and a representative value of 2.6 × 10^−3^ S·cm^−1^ at 65 °C has been found in a reflectin protein (a cephalopod structural protein) [38]. Even if protein-based materials exhibit lower bulk proton conduction than conventional conducting materials, they have outstanding advantages over nonbiological materials for developing bioelectronic devices such as proton transistors. These advantages are biocompatibility, adaptable structure, and tunable transport properties through the amino acid sequence control [39].

As mentioned before, MOFs and SMOFs also exhibit versatile structures, and biocompatibility could be guaranteed by the appropriate selection of both the metal ions and the organic linkers. Porphyrin-based SMOFs, in particular, have several noticeable advantages such as bio-organic linkers and high thermal stability. Porphyrins are naturally occurring macrocyclic compounds such as hemoglobin and chlorophyll, which are highly determinative in the metabolism of living organisms. The stable molecular structure of porphyrin is composed of four pyrrole rings linked via methine bridges, leading to an aromatic character [40,41,42,43,44]. As far as the authors of this article are aware, there are very few examples of porphyrin-based MOFs and SMOFs exhibiting protonic conductivity. For instance, two isoreticular zirconium phenolate porphyrin networks are described by Chen et al. [45] as having “exceptional conductivity” with values of 8.0 × 10^−3^ and 4.2 × 10^−3^ S·cm^−1^, respectively (pelleted sample, under 98% relative humidity at 25 °C). Another example is Cu_2_(CuTCPP) (where H_4_(H_2_TCPP) is 5,10,15,20-tetrakis(4-carboxyphenyl)porphyrin), exhibiting a “high in-plane proton conductivity” of 3.9 × 10^−3^ S·cm^−1^ at 25 °C under 98 % relative humidity [46]. Therefore, porphyrin-based MOFs and SMOFs can be a good compromise for bio-inspired solid-state materials.

However, if real application as proton conductors is desirable for porphyrin-based coordination polymers, conductivity performance should be improved. In order to achieve the latter, the presence of encapsulated molecules of water within the framework is extremely important as it allows the existence of hydrogen bonds. These noncovalent interactions can be formed between a hydrogen atom from an X-H molecular fragment (where X is more electronegative than H) and another atom in the same or a different molecule. Typical X atoms are N, O, and F. As the strength of the hydrogen bond existing in a water dimer is ~5 kcal mol^−1^, thermal fluctuation is enough to explain that it is actually forming and breaking at room temperature [47]. The structural and dynamical properties of hydrogen bonds, along with the molecule reorientation, enhance the high mobility of protons not only in water and aqueous solutions but also among molecules of encapsulated water in coordination polymers. In fact, the term “Grotthuss mechanism” refers to the structural diffusion that the proton transfers from one water molecule to an adjacent one without significant rearrangement of the mass centers [48]. Many studies on hydrogen bonds reveal that the dynamics of the hydrogen bond and the proton migration are highly correlated [49,50,51,52,53,54,55,56,57,58,59,60,61,62,63]. Moreover, proton transportation may be accelerated or delayed when the hydrogen bond network is confined. This is exactly what happens for encapsulated molecules of water along 1D channels, between 2D layered structures, and in 3D pores in solid-state porous materials [64,65,66,67].

**Figure 1:**
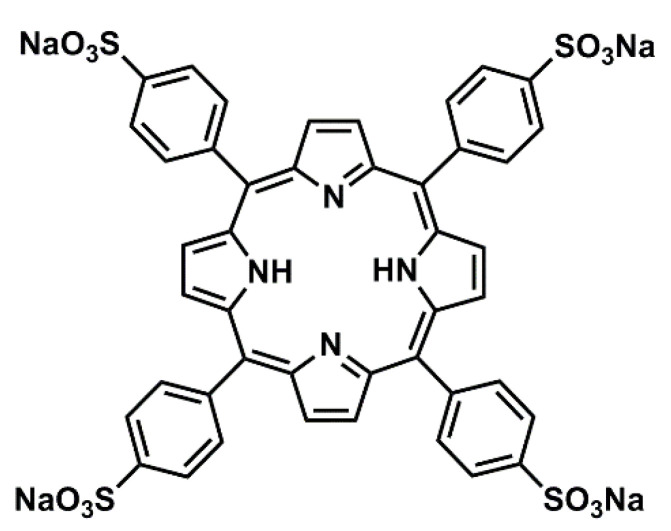
Chemical structure of Na_4_TPPS porphyrin.

In summary, when focusing on developing high-performance proton conductors by using biocompatible solid-state networks porphyrin-based MOFs and SMOFs seem to be a good starting point. However, the selection of metal ions and organic linkers should guarantee the presence of connected cavities in order to confine water molecules for proton transportation purposes. In this sense, in this work we have selected the compound meso-tetraphenylporphine-4,4′,4″,4‴-tetrasulfonic acid tetrasodium salt (Na_4_TPPS) to provide the ligand (TPPS^−4^), also named as *meso*-tetra(4-sulfonatophenyl)porphyrin, which has hydrophilic groups (Figure 1), giving rise to highly stable coordination polymers [68,69]. Compound [H(bipy)]_2_[(MnTPPS)(H_2_O)_2_]·2bipy·14H_2_O (bipy is 4,4′-bipyidine) has been studied by single crystal X-ray diffraction, thermogravimetric analysis (TGA/DSC) and X-ray thermodiffraction (XRTD) measurements [68]. This is an SMOF compound that has been also tested for proton conduction, exhibiting high performance that has been correlated to the presence of a zig-zag chain of confined water within the framework.

## 2. Materials and Methods

All solvents and reagents including meso-tetraphenylporphine-4,4′,4″,4‴-tetrasulfonic acid tetrasodium salt (Na_4_TPPS) 4,4′-bipyridine (bipy) and Mn(NO_3_)_2_·xH_2_O were purchased from Merck.

X-ray diffraction patterns of [H(bipy)]_2_[(MnTPPS)(H_2_O)_2_]·2bipy·14H_2_O were obtained in a Panalytical X’pert CuKα diffractometer 2θ range = 5–70°, step size = 0.015°, exposure time = 10 s per step at room temperature.

Thermogravimetric analyses were carried out using a NETZSCH STA 449F3 thermobalance (Selb, Germany). A crucible containing approximately 10 mg of sample was heated at 5 °C·min^−1^ in the temperature range 30–600 °C. The thermal behavior was also studied using X-ray thermodiffractometry. A Bruker D8 Advance Vantec diffractometer (Cu-Kα radiation) equipped with a variable-temperature stage (Anton Paar HTK2000, Billerica, MA, USA) with a Pt sample holder was used in the experiments. The powder patterns were recorded in 2θ steps of 0.0333° in the 5–38° range, counting for 0.8 s per step and increasing the temperature at 2 °C·min^−1^ from 30 °C to 54 °C. A sealed capillary has been used to maintain 98% relative humidity during measurements.

For the conductivity measurements, the SMOF powder (160 mg) was pressed at 10 tons for 5 min to form a compact disc of 12.92 mm diameter and 0.94 mm thick. The temperature was measured by means of a type K thermocouple in contact with the sample and the relative humidity (RH) was controlled using a saturated aqueous solution of K_2_SO_4_ (~97% RH). The electrical properties were determined for the plane-parallel sample, performing alternating current (AC) complex impedance measurements with a Solartron 1260 Impedance Analyzer (Leicester, UK). The measured frequency range was 10^−1^–10^6^ Hz, with a 5 mV signal amplitude. The behavior of the material was studied in a heating–cooling cycle between room temperature and 70 °C. The impedance diagrams were analyzed and fitted by the Zview 3.0 software. The conductivity values, σ, were calculated using the following expression:

σ = L/A·R,(1)

where L (cm) and A (cm^2^) are the thickness and surface area of the pellet, respectively, and R (ohm) is the resistance of the sample obtained from the intersection of the curve with the real axis in the Nyquist diagram.

## 3. Results and Discussion

### 3.1. Synthesis and Crystal Structure

[H(bipy)]_2_[(MnTPPS)(H_2_O)_2_]·2bipy·14H_2_O (herein after Mn-TPPS) was synthesized as described in [68] obtaining prismatic dark red single crystals. This compound is a SMOF (supramolecular metal–organic framework) consisting of complex ions. The crystal structure shows [(MnTPPS)(H_2_O)_2_]^2−^ anionic monomers with TPPS^4−^ ligands and the Mn^II^ ion octahedrally coordinated to the porphyrin core and axially to two water molecules. The metalloporphyrinic intermonomer space is occupied by [H(bipy)]^+^ cations and crystallization bipyridine molecules, and the interstitial channels are occupied with 14 lattice water molecules per monomer unit [68]. This lattice molecules of water stabilizes the crystal structure by an extensive hydrogen bond system (Table 1) interacting with the sulfonate groups, the [H(bipy)]^+^ cations, the crystallization molecules of bipy, and the coordinated water molecules. The donor–acceptor (O···A) distances of these hydrogen bonds range from 2.540 (4) to 3.010 (2) Å.

The hydrogen bonds are based on the presence of numerous O and N atoms. In fact, atoms from O(1) to O(6) belong to the sulphonate groups, O(7) corresponds to the axially coordinated molecules of water, atoms from O(8) to O(16) correspond to crystallization molecules of water, and atoms from N(3) to N(6) belong to bipy specimens.

**Figure 2:**
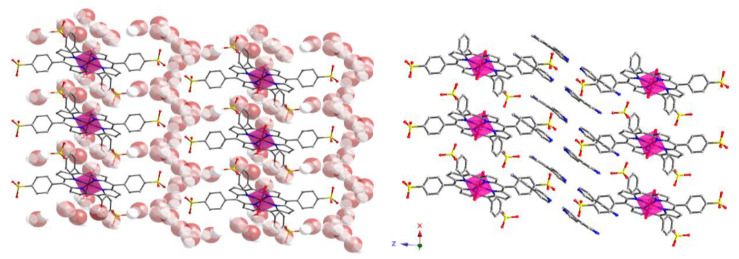
Crystal structure packing for Mn-TPPS. Color code: Mn, pink; C, grey; N, blue; O, red and S, yellow. H atoms have been omitted for clarity: (**left**) bipy units are omitted for clarity, and (**right**) water molecules of crystallization are omitted for clarity.

It is worth noticing that hydrogen bonds involving atoms from O(12) to O(16) give rise to zig-zag chains of water molecules. These chains are interconnected and extend along the [100] direction. As observed in Figure 2, these zig-zag chains are located between the sulfonate groups.

**Figure 3:**
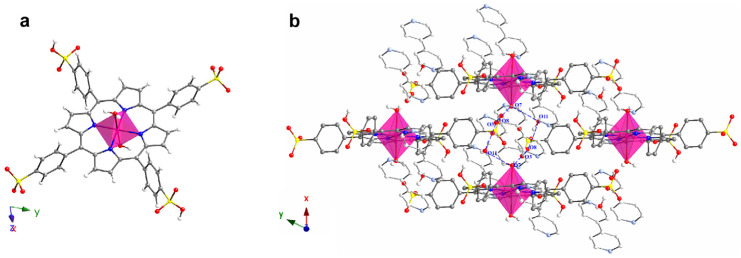
(**a**) Detail of the coordination sphere on the Mn^II^ ion and (**b**) view of the hydrogen bonding system in the crystal structure of Mn-TPPS. Color code: Mn, pink; C, grey; N, blue; O, red; S, yellow and H, white. Hydrogen bonds are marked as blue dashed lines. Porphyrin ring H atoms have been omitted for clarity.

As explained below, the high amount of hydrogen bonds contributes to the high thermal stability of Mn-TPPS. To illustrate the latter, Figure 3 shows a detail of the hydrogen bonding system through the sulfonate groups.

It is worth noticing that the presence of zig-zag non-covalent interactions between molecules of water not only contributes to the stability of the crystal structure but also may exhibit proton conductivity [70,71,72,73].

**Figure 4:**
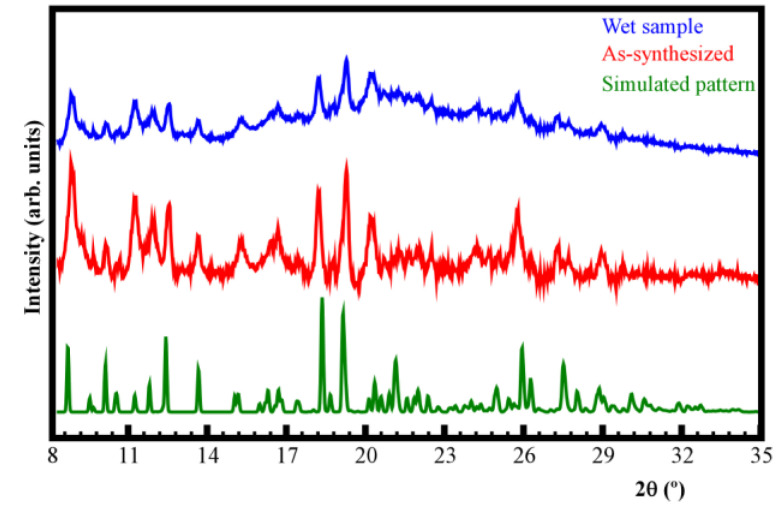
Powder XRD patterns for Mn-TPPS. Simulated pattern (green), as-synthesized (red), and wet sample (blue).

Synthesized single crystals were ground to obtain the bulk sample. Powder XRD analysis confirms that the structural integrity is maintained as the bulk diffraction pattern matches with that simulated from the single crystal X-ray data. Water stability was also evaluated for Mn-TPPS, confirming that the compound remains stable after wetting (Figure 4).

### 3.2. Thermal Analysis

The thermal behavior of the sample was previously evaluated by means of thermogravimetric analysis (TG). The thermogravimetric chart and its interpretation and discussion can be consulted in the work published in 2018 [68]. The stability of the interconnected zig-zag water chains and the amount of water molecules in the compound are closely related to the temperature. From room temperature until 195 °C a continuous weight loss is observed (12.1%) related to the coordination and lattice water molecules. Afterward, until 400 °C crystallization bipyridine molecules are lost (19.7%), between 400 °C and 450 °C the [H(bipy)]+ cations break down (14.3%) and then the degradation of the TPPS units (42.7%) takes place.

**Figure 5:**
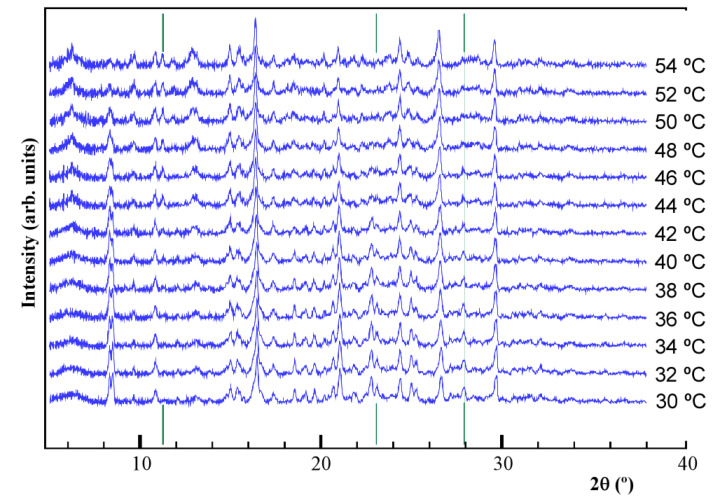
Temperature-dependent XRD patterns for Mn-TPPS under 98% RH conditions. Green line marks at 11.7°, 22.5°, and 27.9° in 2θ have been added.

The temperature-dependent powder X-ray diffraction analysis under humid conditions (98% RH) is shown in Figure 5. As observed, there are slight variations in the patterns corresponding to peaks at 11.7°, 22.5°, and 27.9° in 2θ. In fact, the appearance of a new diffraction peak for the lower angle and the loss of intensity for the other two is attributed to a change or rupture in the lattice water chains, while the porphyrinic entities remain unalterable as there is no change observed in the most intense peaks. As will be discussed later, this fact is directly related to the proton conductivity of this compound.

### 3.3. Proton Conductivity

**Figure 6:**
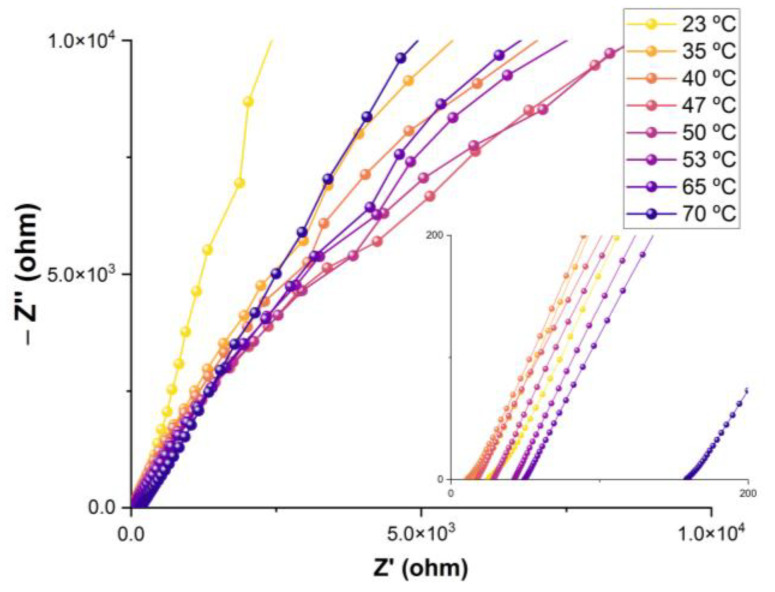
Nyquist plot of the Mn-TPPS sample measured in the temperature range of 23–70 °C at ~97% RH.

The proton conductivity of the sample was evaluated by complex electrochemical impedance spectroscopy (EIS). Figure 6 shows typical Nyquist diagrams obtained at different temperatures with a relative humidity of ~97%. The recorded spectra show the characteristic inclined line associated with proton diffusion processes.

**Figure 7:**
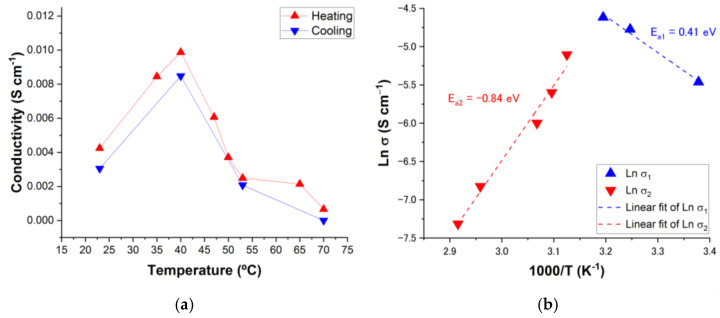
(**a**) Evolution of proton conductivity as a function of temperature at ~97% RH for the Mn-TPPS sample during heating–cooling cycle. (**b**) Arrhenius plot of the proton conductivities in the heating process for Mn-TPPS under ~97% RH conditions.

The resistance at each temperature was estimated from the high-frequency end of the straight line and the ionic conductivity was calculated through expression (1), obtaining the values shown in Figure 7a. The absence of mixed valences that could introduce charge carriers into the compound and the difficulty in developing long-range charge transport pathways allow us to rule out an efficient contribution from electrical conductivity. In ambient humidity (~50% RH) the as-synthesized Mn-TPPS has a conductivity of 2.32 × 10^−5^ S cm^−1^ at 20 °C. The presence of relative humidity allows a higher adsorption of water molecules, also providing a greater mobility of protons through the material and influencing, in this way, the proton conductivity. Thus, the ionic conductivity at 23 °C in the presence of ~97% RH increases to 4.24 × 10^−3^ S cm^−1^, which implies an improvement of two orders of magnitude. As the temperature rises, an increase in conductivity is observed until it reaches a maximum at 40 °C with a value of 9.87 × 10^−3^ S cm^−1^, which is considerably high for MOF and SMOF-type materials [74,75,76]. As temperature increases, there are two contrary effects affecting proton conductivity. On one hand, the mobility of protons increases with temperature and, therefore, this is expected to increase conductivity. On the other hand, the number of protons decreases with temperature as a result of the loss of mass attributed to molecules of water, and this is expected to produce a decrease in conductivity. In the cooling process, the conductivity values recorded are slightly lower than those in the heating process, although they remain in the same order of magnitude. In this way, it is verified that the changes produced in the structure are reversible with temperature.

It is worth noting that, based on the conductivity values obtained for the Mn-TPPS compound (σ > 10^−4^ S cm^−1^), this material can be considered as a superionic conductor or fast ionic conductor [77].

As stated before, upon exceeding 40 °C, the conductivity begins to decrease, this trend being a clear indication of a change in the proton conduction mechanism. Proton conduction assisted by water molecules, as is the case in this work, can be governed by two different mechanisms: the vehicle mechanism and the Grotthuss mechanism [19]. In the first of the mechanisms, proton transport occurs through self-diffusion processes of protogenic species, essentially as proton transporters [78]. In this way, the proton does not migrate isolated through the material but is associated with a “vehicle” such as H_2_O molecules that will act as Brønsted bases. In the Grotthuss mechanism (also known as the proton-hopping mechanism), on the other hand, proton conduction occurs through the network of hydrogen-bonded water molecules [79]. The transfer occurs simultaneously with breaking those hydrogen bonds, transferring the proton, and with the subsequent rearrangement of nearby water molecules. In this way, in the Grotthuss mechanism, the protons are jumping along the path of conduction through processes of protonation and deprotonation of water molecules.

From the conductivity data, the Arrhenius graph can be represented (Figure 7b) and it is possible to calculate the activation energies related to the different transport processes. The activation energy provides an intuitive idea about the type of proton conduction mechanism. In the case of the vehicular mechanism, the activation energy values are markedly higher than in the case of the Grotthuss mechanism, since the proton travels attached to a transporter, which makes the mobile species larger, requiring higher energy. The limit regarding the value of the activation energy is located around 0.4 eV [19,29].

Between room temperature up to 40 °C, the mechanism that must govern the proton conduction throughout the material must be the Grotthuss mechanism through the structural diffusion mechanism (E_a1_ = 0.41 eV). In this way, the transfer of protons takes place through the generation and breaking of hydrogen bonds with the lattice water molecules located between the sulfonate groups that make up the proton-conducting pathway. In any case, it is not possible to totally rule out a certain contribution of the vehicular mechanism [29]. Above 40 °C, the proton conductivity drops drastically to values of 6.66 × 10^−4^ S cm^−1^ at 70 °C. In summary, the Grotthuss mechanism seems to be the most important for the temperature range where the proton mobility governs the conduction, while the vehicle mechanism should operate mainly within the temperature range where the most affected parameter is the number of vehicles (molecules of water). Hurd et al. observed a similar behavior of loss of protonic conductivity with increasing temperature in the Na_3_(2,4,6-trihydroxy-1,3,5-benzenetrisulfonate) MOF, a fact that was justified due to a dehydration process [80].

## 4. Conclusions

Metalloporphyrin-based SMOFs have been identified as potential materials for bioelectronic devices such as proton transistors, as they exhibit the advantages of tunable SMOFs in structures where the ligands have a biological origin. Comprehensive use of the crystal lattice with crystallization molecules of water produces a vast variety of possibilities for proton conduction. For Mn-TPPS, the presence of sulfonate groups as functional groups in the selected ligands is crucial as it produces a structural assembly where zig-zag chains of water molecules are formed through hydrogen bonds. As a result of the Grotthuss mechanism, proton transportation takes place along these chains producing a superprotonic conduction. Therefore, it is demonstrated that through a rational design of the hydrogen bonding network throughout the SMOF cavities, it is possible to obtain new proton conducting systems with huge potential for applications in a wide range of sectors.

## Figures and Tables

**Table 1:** Hydrogen bond parameters for Mn-TPPS (distances in Å and angles in °).

O-H	A(O)	O-H(Å)	H···A(Å)	O···A (Å)	O-H···A (°)	Type
O(7)-H(7A)	O(3) *(−1 + x*, *1 + y*, *z)*	0.82	1.90	2.723 (4)	178	a
O(7)-H(7B)	O(11) *(−1 + x*, *y*, *z)*	0.82	1.94	2.740 (4)	167	a
O(8)-H(8A)	O(7) *(−x*, *2 − y*, *−z)*	0.82	2.00	2.809 (4)	169	b
O(11)-H(11A)	O(8)	0.82	2.03	2.759 (4)	147	b
O(9)-H(9A)	N(3) *(1 − x*, *1 − y*, *1 − z)*	0.83	1.83	2.657 (7)	176	c
O(9)-H(9B)	O(8)	0.83	1.89	2.720 (4)	175	b
O(10)-H(10B)	N(6) *(1 − x*, *1 − y*, *1 − z)*	0.83	2.03	2.823 (5)	160	c
O(10)-H(10A)	O(12)	0.83	1.87	2.692 (3)	177	b
O(12)-H(12A)	O(13)	0.82	1.83	2.540 (4)	145	d
O(12)-H(12A)	O(14)	0.82	1.97	2.766 (4)	163	d
O(13)-H(13A)	O(12)	0.83	2.07	2.540 (4)	123	d
O(13)-H(13B)	O(15)	0.82	2.22	2.787 (5)	126	d
O(15)-H(15A)	O(13)	0.82	2.42	2.787 (5)	108	d
O(15)-H(15A)	O(16)	0.82	2.19	2.936 (5)	150	d
O(12)-H(12B)	O(2)	0.82	1.94	2.752 (4)	169	e
O(14)-H(14B)	O(6) *(x*, *−1 + y*, *z)*	0.82	2.28	3.010 (2)	148	e
O(14)-H(14B)	O(6B) *(x*, *−1 + y*, *z)*	0.82	2.01	2.770 (3)	153	e
O(16)-H(16A)	O(5)	0.83	2.26	2.667 (2)	111	e
O(16)-H(16A)	O(5B)*(1 + x*, *−1 + y*, *z)*	0.83	2.35	2.820 (3)	117	e
O(6)-H(6)	O(15) *(x*, *1 + y*, *z)*	0.86	2.35	2.745 (2)	109	e
O(6)-H(6B)	O(14) *(x*, *1 + y*, *z)*	1.15	1.99	3.010 (2)	145	e
O(6)-H(6B)	O(15) *(x*, *1 + y*, *z)*	1.15	2.16	2.745 (2)	108	e
N(5)-H(1N)	N(4)	1.28	1.46	2.741 (7)	171	f

Types according to the participants: a. Coordinated molecules of water, out-of-chain crystallization molecules of water, and sulfonate groups in TPPS^−4^. b. Out-of-chain crystallization molecules of water, in-chain crystallization molecules of water, and sulfonate groups in TPPS^−4^. c. Bipy entities and out-of-chain coordinated molecules of water. d. In-chain crystallization molecules of water. e. In-chain crystallization molecules of water and sulfonate groups in TPPS^−4^. f. Bipy entities.

## Data Availability

The data presented in this study are available on request from the corresponding author. The data are not publicly available due to privacy.
